# *Chroesthes* (Acanthaceae) in Peninsular Malaysia, including a new species from Kelantan and a new record from Terengganu

**DOI:** 10.3897/phytokeys.174.62023

**Published:** 2021-03-12

**Authors:** Siti-Munirah Mat Yunoh

**Affiliations:** 1 Forest Research Institute Malaysia, 52109, Kepong, Selangor, Malaysia Forest Research Institute Malaysia Kepong Malaysia

**Keywords:** Conservation status, revision, taxonomy

## Abstract

*Chroesthes* is a small genus that includes three species from Peninsular Malaysia: *Chroesthes faizaltahiriana* Siti-Munirah **sp. nov.**, *C. lanceolata* (T. Anderson) B.Hansen and *C. longifolia* (Wight) B.Hansen. *Chroesthes faizaltahiriana*, recently discovered in the State of Kelantan, is described and illustrated. This species is similar to the common species *C. longifolia*, but is distinguished mainly by its inflorescence type, calyx shape and its flowers being bright orange instead of dark purple internally. *Chroesthes lanceolata* is a new record for Peninsular Malaysia and has only been collected once. Following the IUCN Red List Categories and Criteria, these three species are assessed (national scale assessment) as Critically Endangered (*C. faizaltahiriana* and *C. lanceolata*) and Least Concern (*C. longifolia*).

## Introduction

The small genus *Chroesthes*Benoist (1927), Acanthaceae, currently consists of three species distributed in South China, Indo-China and Malaysia (Hansen 1983; Govaerts 1999; POWO 2019). In the current classification of the family Ranunculaceae, it is placed within the putative members of tribe Barlerieae (Scotland and Vollesen 2000; McDade et al. 2008; Champluvier and Darbyshire 2012). This genus can be distinguished from other genera of Acanthaceae by the structure of the calyx and corolla, the presence of bicalcarate anthers, i.e. anther thecae spurred at the base or awned anther thecae and are diagnostic within Barlerieae, for which quincuncial aestivation is diagnostic and seeds are shortly pubescent (Hansen 1983; Hu et al. 2011; Darbyshire et al. 2019). The three species of *Chroesthes* are distributed as follows: *Chroesthes lanceolata* (T. Anderson) B.Hansen occurs in Myanmar, N Thailand, N Laos, N Vietnam SW China (Yunnan) and Peninsular Malaysia (Hansen 1983; Ummul-Nazrah et al. 2011); *Chroesthes bracteata* (J.B. Imlay) B.Hansen occurs in SE Thailand (Hansen 1983); and *Chroesthes longifolia* (Wight) B.Hansen is endemic in Peninsular Malaysia (Hansen 1983).

The Forest Research Institute Malaysia (FRIM) launched the Peninsular Malaysia Flora Project (FPM) in 2005, which aims to provide a more complete and up-to-date account of the national flora (Middleton et al. 2019). To achieve this goal, the FRIM flora team continues to conduct vegetation surveys throughout Peninsular Malaysia, especially in unexplored and ecologically-significant areas. Through this fieldwork, the number of species encountered within the Flora region has increased. A few new species are discovered every year and published in journals. For example, many new fairy lantern species (*Thismia*) have been described in recent years, including *Thismia kelantanensis* Siti-Munirah, *T. domei* Siti-Munirah and *T. terengganuensis* Siti-Munirah (Siti-Munirah 2018; Siti-Munirah and Dome 2019). Moreover, new gesneriad species have also been described, including *Codonoboea norakhirrudiniana* Kiew, *C. rheophytica* Kiew, *C. sallehuddiniana* Lim (Kiew and Lim 2019) and, most recently, *Codonoboea kenaboiensis* Syahida-Emiza, Sam & Siti-Munirah and *C. ruthiae* Syahida-Emiza, Sam & Siti-Munirah have been described (Syahida-Emiza et al. 2020).

During a recent botanical survey at Berangkat Forest Reserve (FR), Kelantan, an upright shrub with an unusual bright orange corolla colour was encountered growing in a patch of forest, under shade beside an old logging road. Its morphological characteristics, including its stamen type, indicated that it belongs to the genus *Chroesthes*, but is unmatched amongst the three species currently recognised. This new species is described here as *Chroesthes faizaltahiriana* Siti-Munirah, which brings the total species for *Chroesthes* in the world to four. It is also an additional endemic species in Peninsular Malaysia. With this recent discovery, an account of the genus *Chroesthes* in Peninsular Malaysia is provided, including the key to *Chroesthes* of the world; however, only the Malaysian species are considered in the remainder of the treatment.

## Materials and methods

Specimens of *Chroesthes* species from Peninsular Malaysia, held in the herbaria at Kepong Herbarium (**KEP**) and Singapore Botanic Gardens Herbarium (**SING**), were examined. All cited specimens were observed by the author. The study of the new species was based on material collected by the author on 26 February 2020 from the only populations found during a botanical trip to Berangkat FR, Gua Musang, Kelantan. The specimens were pressed as herbarium specimens and an inflorescence was also preserved as a spirit collection. Morphological characters were studied using a stereomicroscope and high-resolution macro photography. Measurements were taken from living plants and herbarium material. The specimen details were compared with original drawings and descriptions given in the protologues for each species of the genus *Chroesthes* and also with information gathered from the relevant literature (Wight 1850; Clarke 1908; Ridley 1923; Hansen 1983, Hu et al. 2011). The conservation status assessments were made with reference to the Categories and Criteria of IUCN (2019).

## Taxonomy

### Chroesthes

Taxon classificationPlantaeLamialesAcanthaceae

Benoist

B3BD018C-4406-5C7B-B32B-964B3AC78F44

Bull. Mus. Natl. Hist. Nat. 33: 107. 1927, in Fl. Gen. I.C. 4: 684. 1935; Hansen, Nordic J. Bot. 3: 209. 1983; Hu, J.C., Deng, Y.F., Daniel, T. & Wood, J.R.I. Acanthaceae. In: Wu, Z.Y., Revan, P. & Hong, D.Y. (Eds.) Flora of China 19: 472. 2011.

#### Description.

Shrubs; cystoliths present. ***Leaves*** opposite, petiolate; leaf blade margin entire; subisophyllous or anisophyllous. ***Inflorescences*** terminal thyrses (the thyrses are branched (then paniculiform) or unbranched (then racemoid); bracts and bracteoles greenish. ***Calyx*** unequally five-lobed: posterior lobe largest, two lateral lobes smaller than two anterior lobes. ***Corolla*** tube basally cylindrical, expanded distally into a throat; limb two-lipped, upper lip two-lobed, lower lip three-lobed; lobes quincuncial in bud; four stamens, connate to the corolla, not connate to one another, posterior pair shorter than anterior pair, inserted at the base of the corolla throat; anthers bi-thecous; thecae parallel, inserted at different heights, dorsally pubescent, base of each theca spurred; ovary with two ovules per locule; style basally sparsely pubescent; stigma capitate. ***Capsule*** stipe absent or barely present; retinacula present. ***Seeds*** compressed, brownish, shortly pubescent.

#### Distribution.

China, Laos, Malaysia, Myanmar, Thailand, Vietnam. Three species in Malaysia.

#### Ecology.

Lowland dipterocarp forest to upper hill dipterocarp forest.

### Key to species of *Chroesthes*

**Table:** 

1	Bracts approximately half the length of the calyx	***C. lanceolata***
–	Bracts approximately as long as the calyx	**2**
2	All calyx lobes narrow, linear	*C. bracteata* (only in Thailand)
–	Posterior and anterior calyx lobes elliptic to elliptic-lanceolate, the lateral lobes linear	**3**
3	Inflorescence terminal branched; corolla entirely dark purple to purplish-red or sometimes white externally	***C. longifolia***
–	Inflorescence terminal unbranched; corolla yellow to dark orange	***C. faizaltahiriana***

### Chroesthes faizaltahiriana

Taxon classificationPlantaeLamialesAcanthaceae

Siti-Munirah sp. nov.

975DDF77-A388-59E4-9FB3-EBD5CE8F804D

urn:lsid:ipni.org:names:77215721-1

Figs 1 , 2 , 3

#### Diagnosis.

*Chroesthes faizaltahiriana* most closely resembles *C. longifolia*; however, it differs in its inflorescence type, the presence of a terminal raceme not branching (vs. terminal raceme branching) and posterior lobe size ratio 1:4 (vs. 1:2) and corolla length 4.5–5.5 cm (vs. 2–3 cm) and in the corolla tube and lobes being entirely bright yellow to dark orange (vs. entirely dark purple, purplish-red or occasionally white externally) and other significant characters (see Table 1).

**Table 1.** Morphological comparison between *Chroesthes faizaltahiriana* and *C. longifolia*.

Character	*C. faizaltahiriana*	*C. longifolia*
Habit		
*Height* (m)	0.7–1	1–2.5
*Stem*	Unbranched	Branching
Inflorescence		
*Type*	Terminal raceme not branching (single)	Terminal raceme always branching (always two)
*Length* (cm)	8–13	up to 25
Flowers		
*Bract* (mm)	20–30 × 2–5	15–20 × 4.5–6
*Shape*	Narrowly lanceolate	Lanceolate
*Bracteoles* (mm)	10–15 × 1–2	10 × 3
*Calyx length* (cm)	2–3	1.6–1.8
*Posterior lobe* (mm)	30 × 7	18 × 8–9
*Anterior lobe* (mm)	22 × 2–2.5	16 × 5–6
*Lateral lobe* (mm)	20 × 1	10–12 × 0.5
*Calyx colour*	Completely always greenish	Green or green to purplish (especially purplish at apex)
*Total corolla length* (cm)	4.5–5.5	2–3
*Corolla colour*	Entirely yellow to dark orange: pale yellow externally, bright orangish-yellow with darker spots internally	Entirely dark purple to purplish-red or sometimes white externally
Filament length (cm)		
*Longer pair*	1.5	1
*Shorter pair*	1.2	0.8

**Figure 1.**
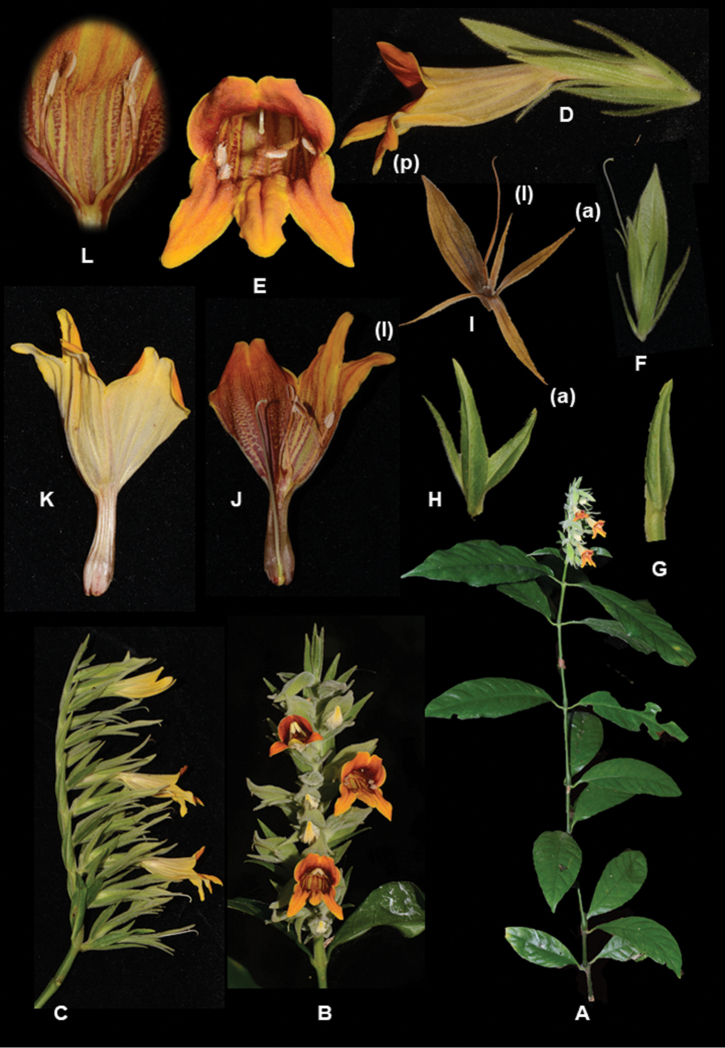
*Chroesthes faizaltahiriana* Siti-Munirah **A** habit of the whole plant **B** inflorescence (front view) **C** inflorescence (side view) **D** flower with bracteoles and calyx **E** perianth lobes (flower from front view) showing the anthers **F** calyx and bracteoles **G** bract **H** bracteoles **I** calyx (a) anterior, (l) lateral, (p) posterior lobes (photo of a dry specimen) **J** corolla (inner view) **K** corolla (outer view) **L** stamens. (All photos by Siti-Munirah MY).

#### Type.

Malaysia. Peninsular Malaysia: Kelantan, Gua Musang Distr., Berangkat FR, ca. 822 m alt., 26 Feb 2020, *Siti-Munirah, FRI 91215* (holotype KEP!, barcode KEP 280001).

#### Description.

Shrubs 0.7–1 m high. ***Stems*** terete, erect, not branched, surface glabrous, diameter ca. 2 mm, swollen at nodes. ***Leaves*** opposite; petiole 0.2–1 cm long; straight or twisted (makes the leaf arrangement look decussate); leaf blades elliptic, lanceolate to oblanceolate, 4.5–19 × 1.5–5.5 cm, both surfaces glabrous, lateral veins ca. 8–10 on each side of mid-vein, base attenuate to cuneate, margin entire, apex acute to acuminate. ***Inflorescence*** a terminal raceme (unbranched), up to 13 cm long; flowers secund (one bract at each node being sterile, the other bracts subtending each flower), ca. 10-flowered; glandular-pubescent on most parts; peduncles ca. 1 cm; bracts narrowly lanceolate, 20–30 × 2–5 mm, apex acute, glandular-pubescent, conspicuously 1-nerved; bracteoles narrowly lanceolate, 10–15 × 1–2 mm; pedicel short, ca. 1–2 mm long. ***Calyx*** 2–3 cm long, posterior lobe lanceolate, 30 × 7 mm, 3–5-nerved, apex acuminate; two anterior lobes, elliptic-lanceolate, 22 × 2–2.5 mm, 1-nerved, apex acuminate; two lateral lobes, linear-lanceolate, 20 × 1 mm, 1-nerved; all glandular-pubescent on both sides; all greenish. ***Corolla*** bilabiate, orange-yellow, ca. 4.5–5.5 cm long; outer surface pale yellow, glandular-pubescent; inner surface bright orange-yellow to orange with dark orange spots (or stripes), glabrous; tube with cylindrical basal portion ca. 2 cm long, expanded throat ca. 1.5 cm long; upper lip shortly two-lobed (8–9 mm long), lower lip deeply three-lobed (8–10 mm long). Stamens 4, didynamous, included in the throat, inserted at the base of the inflated part of the corolla, longer pair with filaments ca. 1.5 cm long, shorter pair with filaments ca. 1.2 cm long, all filaments with sparse glandular trichomes on the surface; anther thecae ca. 1–2 mm long, basal spur pointed, surface cover with simple trichomes. Pistil whitish-green; ovary ovoid, 1.5–2 mm long, apex pubescent; style ca. 3.2 cm long, pilose below, glabrous above; stigma subcapitate, minutely bilobed. Fruit not known.

**Figure 2.**
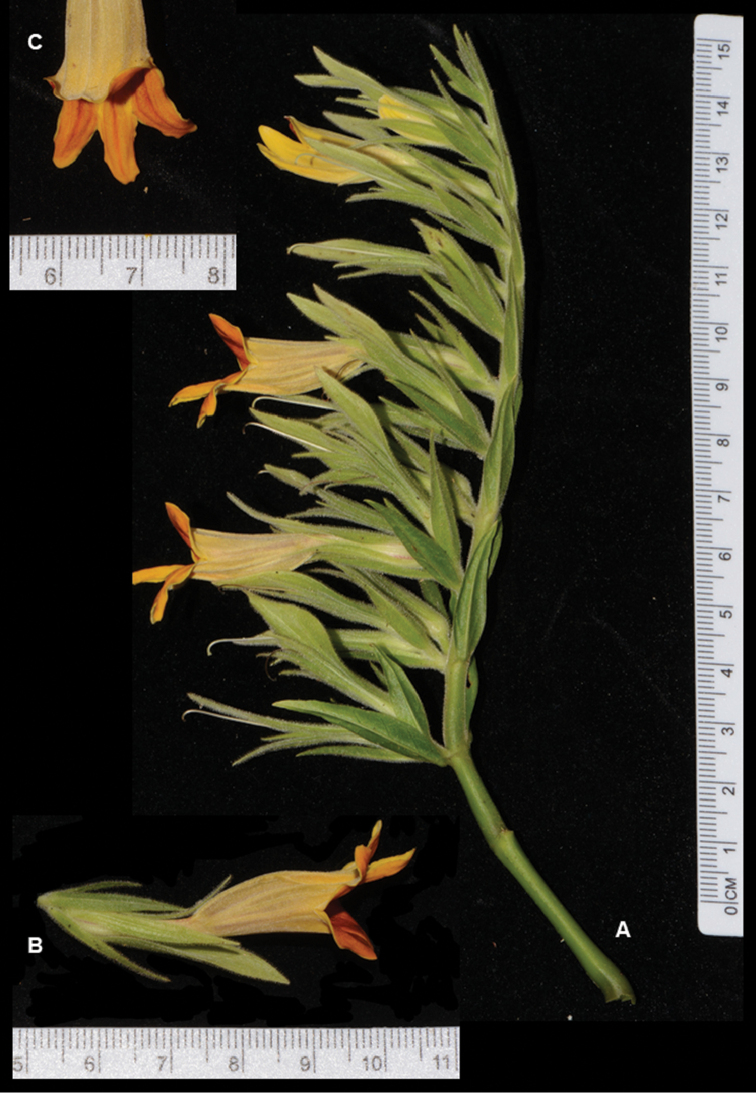
*Chroesthes faizaltahiriana* Siti-Munirah with scale **A** inflorescence **B** one floral unit with calyx and bracteole **C** corolla lobes (All photos by Siti-Munirah MY).

**Figure 3.**
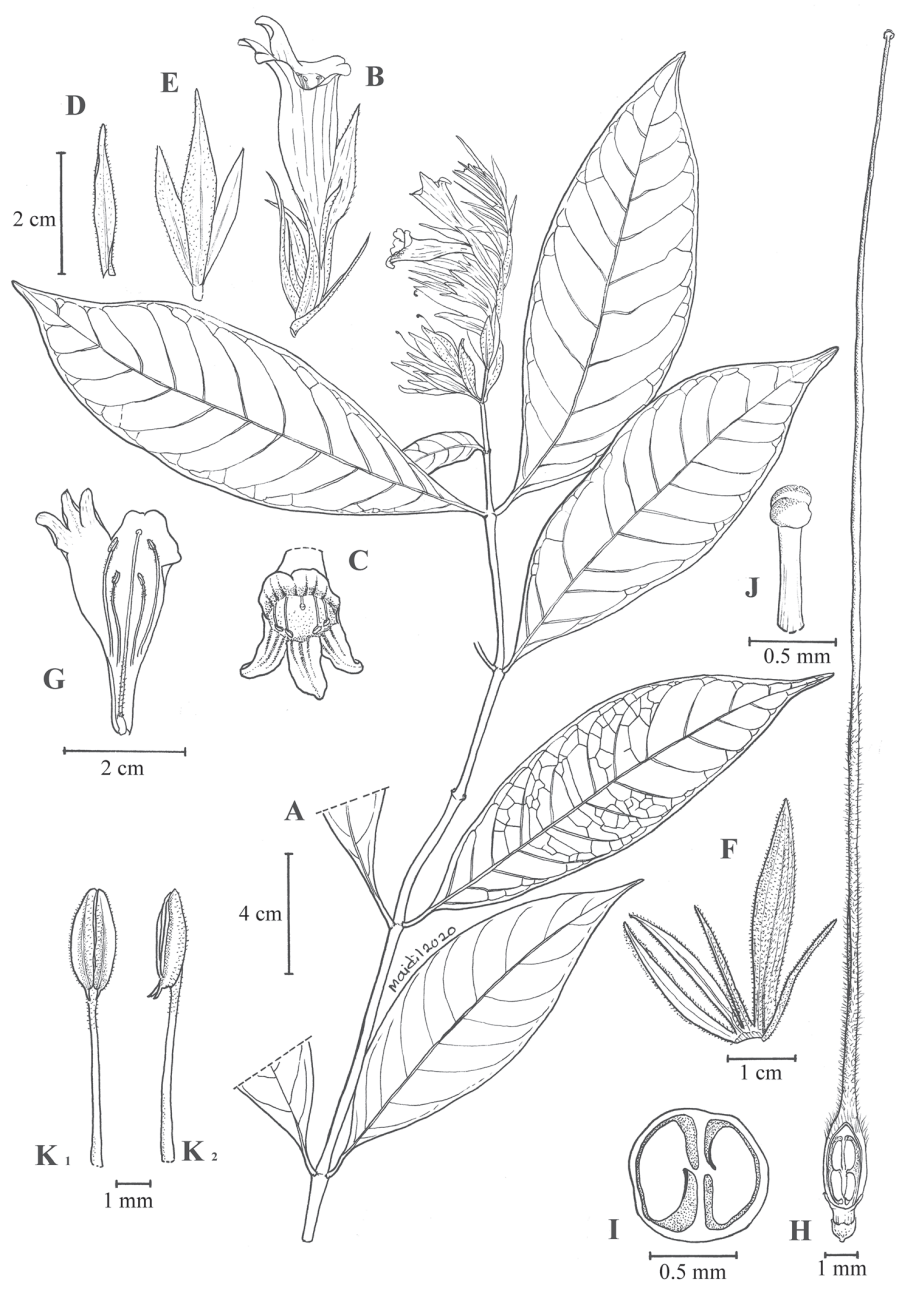
*Chroesthes faizaltahiriana* Siti-Munirah **A** habit **B** a flower with bracteoles and calyx front side view **C** perianth lobes (flower from front view) **D** bract **E** bracteole **F** calyx **G** corolla (inner view) showing the stamens and pistil **H** pistil **I** cross-section of the ovary **J** stigma **K1** anthers front view **K2** anthers side view. All from *FRI 91215*, drawn by Mohamad Aidil Noordin).

#### Distribution.

**Map 1.**
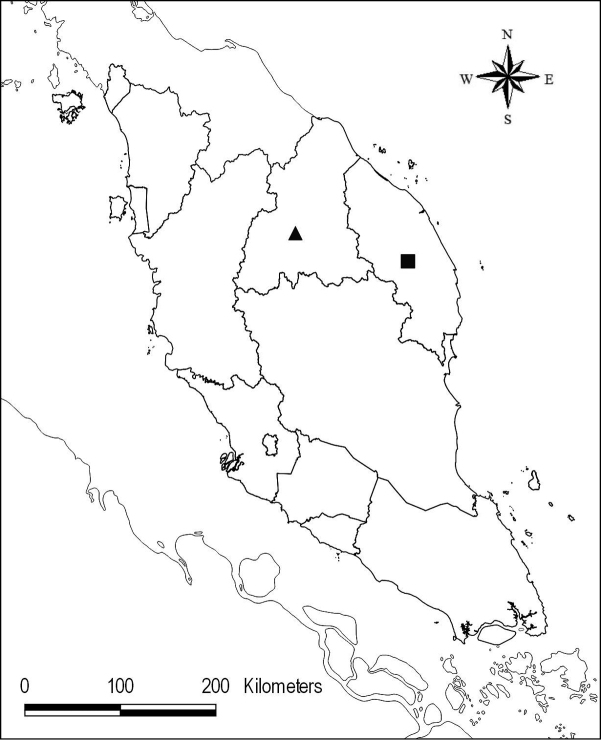
Distribution of *Chroesthes faizaltahiriana* (▲) and *C. lanceolata* (■) in Peninsular Malaysia.

Endemic to Peninsular Malaysia, Kelantan. Currently known only from the type collection from Berangkat FR, 5°07'55.5"N, 101°55'28.5"E (Map 1).

#### Ecology.

**Figure 4.**
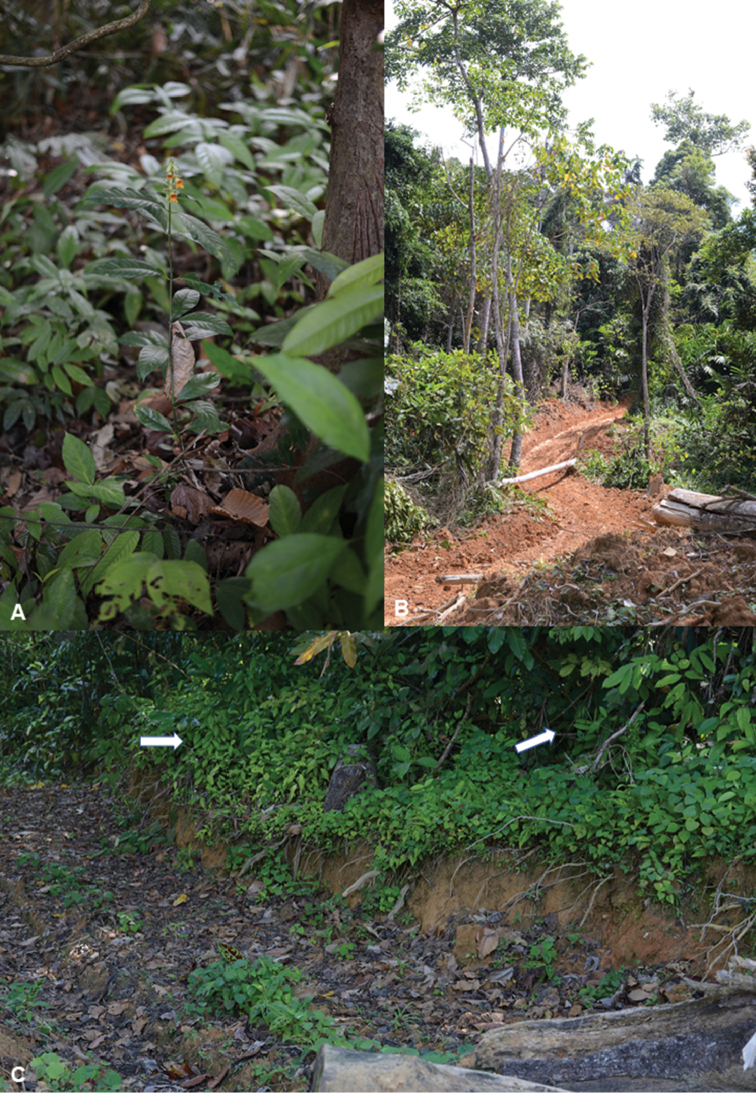
Habitat of *Chroesthes faizaltahiriana* Siti-Munirah **A** plant in its original habitat **B** new logging road beside the population **C** plant found on the bank of an old logging road (white arrow). (All photos by Siti-Munirah MY).

*Chroesthes faizaltahiriana* is found in upper hill dipterocarp forests under shade at 822 m elevation. It was found flowering in February in patches of unlogged forest beside a logging road (Fig. 4).

#### Etymology.

*Chroesthes faizaltahiriana* is dedicated to Mohd Faizal Mat Tahir (known as Faizal Tahir), the husband of Siti-Munirah for his strong support in many ways towards the author’s botanical work.

#### Conservation status.

Critically Endangered B2 ab(ii,iii). Following the IUCN Red List Categories and Criteria (IUCN 2019), this species is assessed as critically endangered because it is only known from one locality and AOO is less than 10 km^2^. Furthermore, about eight individuals were observed (all at the flowering stage). The collection locality is within the forest reserve, in an area of disturbed forest near a logging road at an elevation of 822 m. The status of the population is uncertain because the active selective logging activity within the forest reserve is ongoing. It is certainly not within a Totally Protected Area. During the 3-day visit to Berangkat FR, no other populations of *C. faizaltahiriana* were found. A further survey is needed to obtain more information that could estimate and determine the population size of this species.

#### Notes.

**Figure 5.**
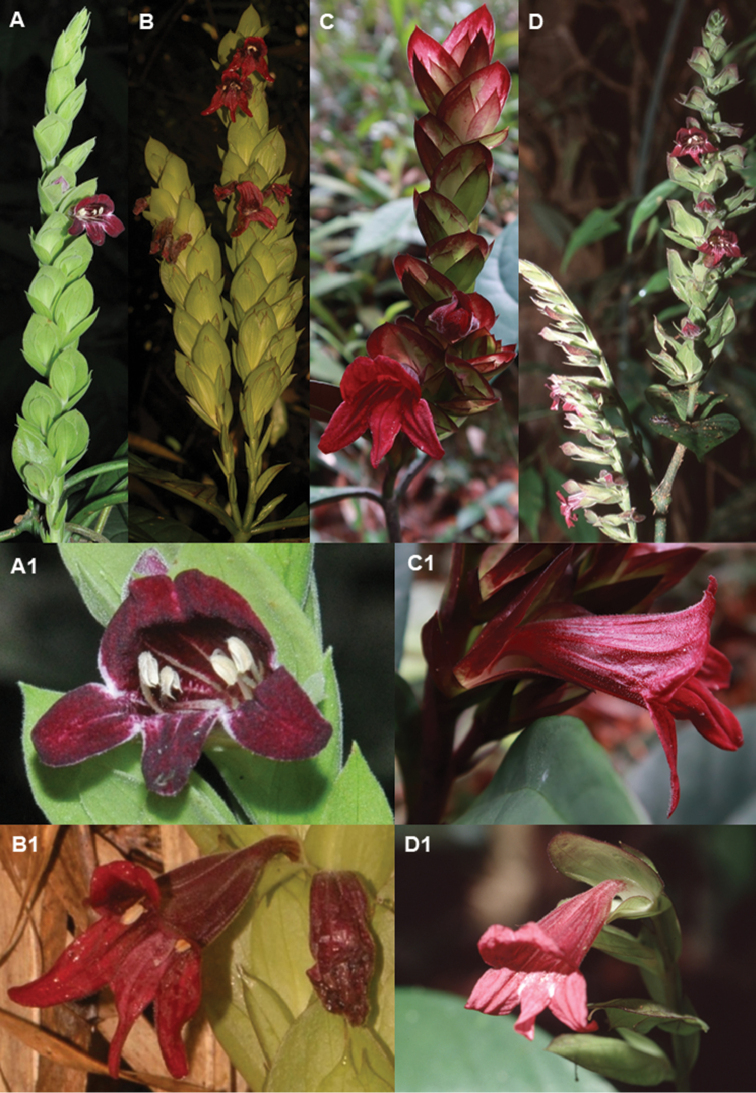
Inflorescence of *Chroesthes longifolia* from four localities **A, A1** Pasoh FR (Negeri Sembilan) **B, B1** Tembat FR (Terengganu) **C, C1** Nerus FR (Terengganu) **D, D1** Gn. Aais FR (Pahang). (Photos: **A, A1** Yao TL **B, B1** Siti-Munirah MY **C, C1** Imin K **D, D1** Sam YY).

Based on the general morphology of this plant, *C. faizaltahiriana* is close to *C. longifolia*, which was previously the only known species of *Chroesthes* in Peninsular Malaysia. However, a detailed comparison has shown that its inflorescence type is entirely different (Fig. 5, Table 1). Additionally, its phytogeography is also different when compared to *C. faizaltahiriana*, which is endemic to Gunung Berangkat (Kelantan) in upper hill dipterocarp forest (822 m a.s.l.), while *C. longifolia* is a widely distributed species inhabiting lowland dipterocarp forest. Based on herbarium specimen collections of *C. longifolia* at Kepong Herbarium (KEP) and the Singapore Herbarium (SING), it has been recorded in Kedah, Perak, Kelantan, Terengganu, Pahang, Selangor, Negeri Sembilan, Melaka and Johor. *C. faizaltahiriana* is a distinctive species amongst *Chroesthes* due to its flower colour. It is the only *Chroesthes* species with bright orange flowers internally. The flowers of *C. longifolia* are entirely dark purple to deep purplish-red (occasionally white externally), while the flowers of *C. lanceolata* are white, spotted pink to purple and those of *C. bracteata* are reported as pale pink, spotted with red.

### Chroesthes lanceolata

Taxon classificationPlantaeLamialesAcanthaceae

(T. Anderson) B. Hansen

4EDCAE03-1A63-5C62-AC1F-EDB6149BC2EF

Fig. 6

Chroesthes lanceolata (T. Anderson) B. Hansen, Nordic J. Bot. 3: 209. 1983; Hu, J.C., Deng, Y.F., Daniel, T. & Wood, J.R.I. Acanthaceae. In: Wu, Z.Y., Revan, P. & Hong, D.Y. (Eds.) Flora of China 19: 472. 2011. Basionym: Asystasia lanceolata T. And., J. Linn. Soc., Bot. 9: 524. 1867. *Type*: Myanmar, Pegu, Thaungyin, *Brandis s.n.* (holotype CAL).

#### Description.

Shrubs 0.5–3 m tall, anisophyllous. ***Stems*** terete, slender, rarely branched, glabrous. ***Leaves*** petiole 1–2.5 cm; leaf blade elliptic to oblanceolate to lanceolate, 10–16 × 3–7 cm, both surfaces glabrous, secondary veins 6–9 on each side of mid-vein, base cuneate, margin entire or sub-sinuate, apex acuminate. ***Inflorescence*** thyrses 3–7 cm; cymes sessile, 1–3-flowered; peduncles ca.2 cm; bracts elliptic to broadly lanceolate, 3–9 × 1–3 mm, apex acute, glandular-pubescent; bracteoles narrowly elliptic to broadly lanceolate, 4–9 × 0.7–1.2 mm; pedicel 1–5 mm long. ***Calyx*** 1–1.6 cm, outside glandular-pubescent; posterior lobe lanceolate, ovate or subelliptic; anterior lobes connate to two-thirds of their length. lateral lobes linear-lanceolate. ***Corolla*** white with pink or purple spots, ca. 2.5 cm, outside pubescent; tube basal portion ca. 9 mm, throat ca. 1.5 cm; upper lip two-lobed; lower lip three-lobed. Stamens 4, included in throat; filaments 1–1.2 cm, glabrous; anther thecae 2.1–2.3 mm, pubescent at the apex and along sides, basal spur pointed; ovary apex pubescent; style ca. 2.5 cm. Capsule subellipsoid to obovoid, 1.2–1.6 cm, glabrous or only at apex pubescent, four-seeded. Seeds subcircular in outline.

#### Distribution.

Myanmar, N Thailand, N Laos, N Vietnam, SW China (Yunnan), Malaysia. In Peninsular Malaysia, recorded from one specimen collected from the trail to Gunung Padang, Ulu Brang, Terengganu in 2010 (FRI 66129) (Map 1).

#### Ecology.

In Peninsular Malaysia, found in a lowland dipterocarp forest at 473 m a.s.l., under a canopy near a small river. (Trail to Gunung Padang).

#### Conservation status.

Critically Endangered B2 ab(ii). Following the IUCN Red List Categories and Criteria (IUCN 2019), this species is assessed as critically endangered at a national level in Peninsular Malaysia because it is currently known from only one specimen in one locality. It is certainly a very rare species. The forest area is lowland dipterocarp forest which was previously logged in the past and it is not a Totally Protected Area. Globally, its conservation status possibly lists it as least concern (LC), by its wide range distribution.

#### Specimen examined.

Terengganu: Hulu Terengganu, Ulu Brang, Ummul-Nazrah et al. FRI 66129 (KEP).

**Figure 6.**
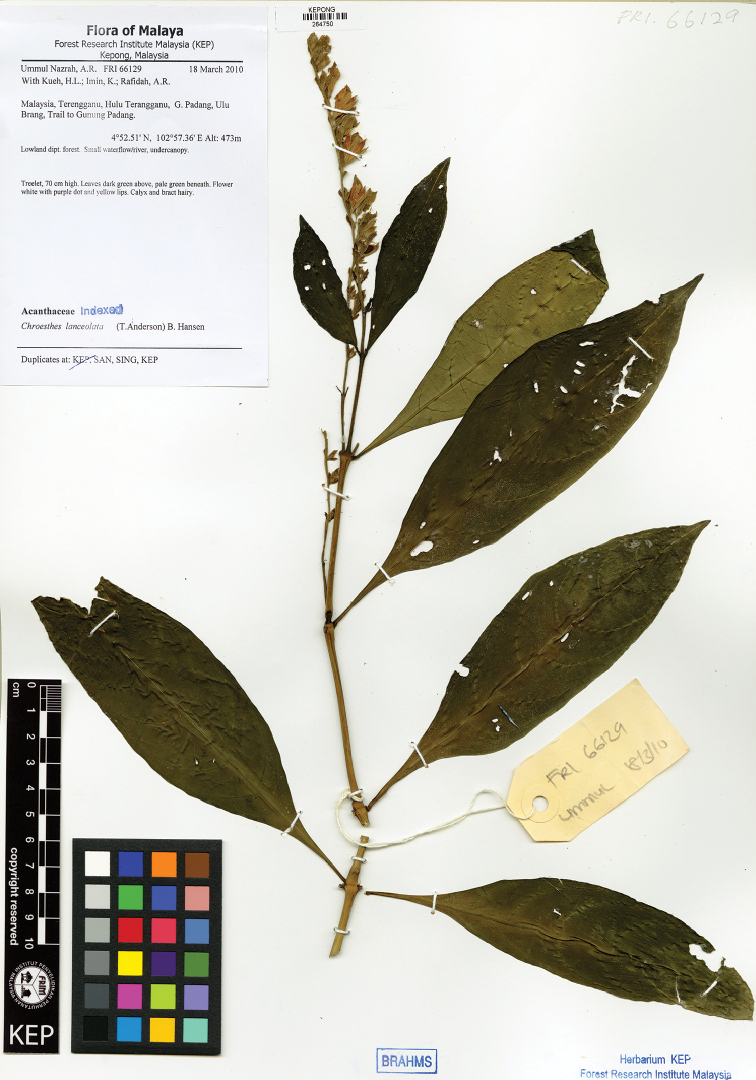
*Chroesthes lanceolata* (T. Anderson) B. Hansen collected from Gunung Padang, Terengganu. (FRI 66129).

### Chroesthes longifolia

Taxon classificationPlantaeLamialesAcanthaceae

(Wight) B. Hansen

72AF757E-1E52-595F-B132-D6BEDAE46FE5

Figs 7 , 8

Chroesthes longifolia (Wight) B. Hansen Nordic J. Bot. 3: 210. 1983. Basionym: Lepidagathis longifolia Wight, Icones Plantarum Indiae Orientalis 4 (4): 8–9, Pl: t.1564. 1850; Ridley, The Flora of the Malay Peninsula 2: 587. 1923. *Type*: Malaysia, Malacca, *Griffith s.n.* (lectotype K).

#### Description.

Shrub 1–2.5 m high. ***Stem*** branches terete, glabrous. ***Leaves*** with petiole 0.5–2 cm long; lamina lanceolate, 16–24 × 3.4–8 cm, glabrous, lateral nerves up to 14 pairs, base attenuate, margin entire, apex acuminate. ***Inflorescence*** terminal raceme branching, up to 25 cm long; flowers secund, peduncles up to ca. 2.5 cm; one bract at each node being sterile, the other bract subtending one flower; bracts lanceolate, 15–20 × 4.5–6 mm, base obtuse, apex acuminate, glandular-pubescent, nerves conspicuous; bracteoles lanceolate, 10 × 3 mm, as bracts; pedicel very short. ***Calyx*** 1.6–1.8 cm long, posterior and anterior lobes elliptic-lanceolate, acuminate, conspicuously nerved, one posterior lobe, elliptic-lanceolate, 18 × 8–9 mm, 3–5 nerved/conspicuously nerved, apex acuminate; two anterior lobes, elliptic-lanceolate, 16 × 5–6 mm, 1-nerved/conspicuously nerved, apex acute; two lateral lobes, linear-lanceolate, 10–12 × 0.5 mm, one-nerved; all glandular-pubescent on both sides; lateral segments linear, one-nerved, glandular-pubescent on both sides; calyx greenish to purplish on the upper part or at the apex. ***Corolla*** bilabiate, dark purple-maroon (or claret), 2–3 cm long; outer surface dark purple, dark maroon, claret, sometimes turning white, glandular-pubescent outside; inner surface, dark purple, dark maroon, claret, sometimes whitish on nerves and base, glabrous; narrow part of tube 0.6 cm long, inflated part 1 cm; upper lip shortly emarginate, ca. 3–4 mm long, lower lip deeply trifid, ca. 5 mm long. Stamens 4, inserted at the base of the inflated part of the corolla; longer pair filament 1 cm, shorter pair filament 0.8 cm, filaments 0.8–1 cm long, glabrous, sometimes with glandular trichomes; anthers thecae ca. 1 mm long, glandular-pubescent along the back, bicalcarate at the base. Pistil whitish-green, stigma capitate; ovary glabrous; style ca. 1.4 cm long, pubescent. Capsule ca. 1.5 cm long, glabrous.

**Figure 7.**
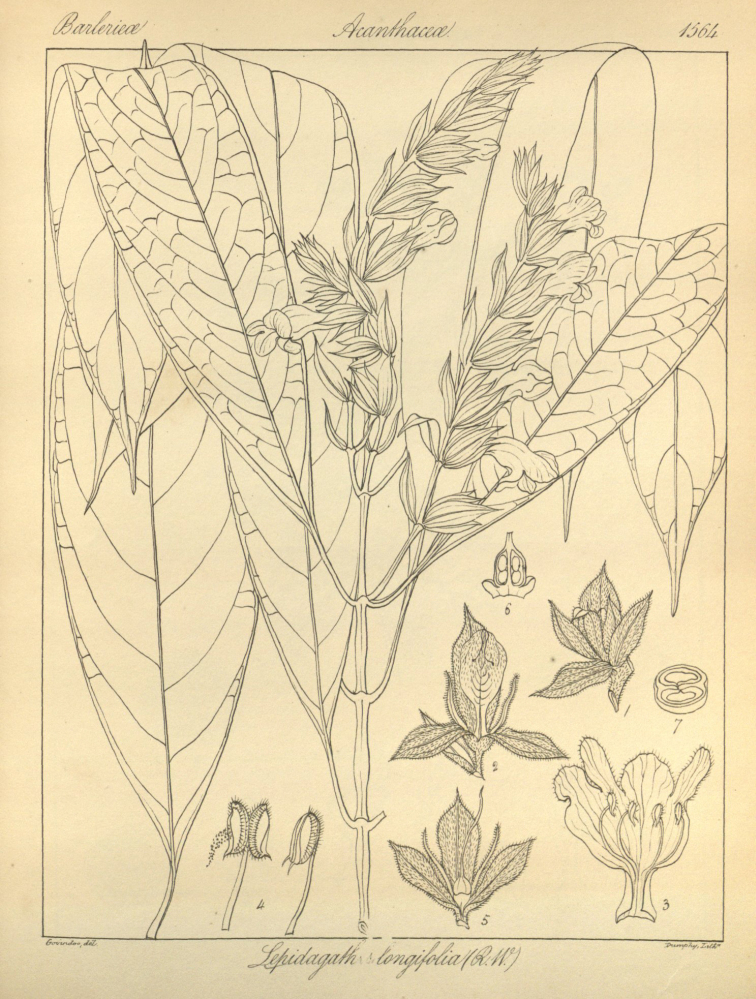
*Chroesthes longifolia* (Wight) B. Hansen. The drawing from the original protologue. Source: Icon. Pl. Ind. Orient. 4 (1850) t. 1564.

#### Distribution.

**Map 2.**
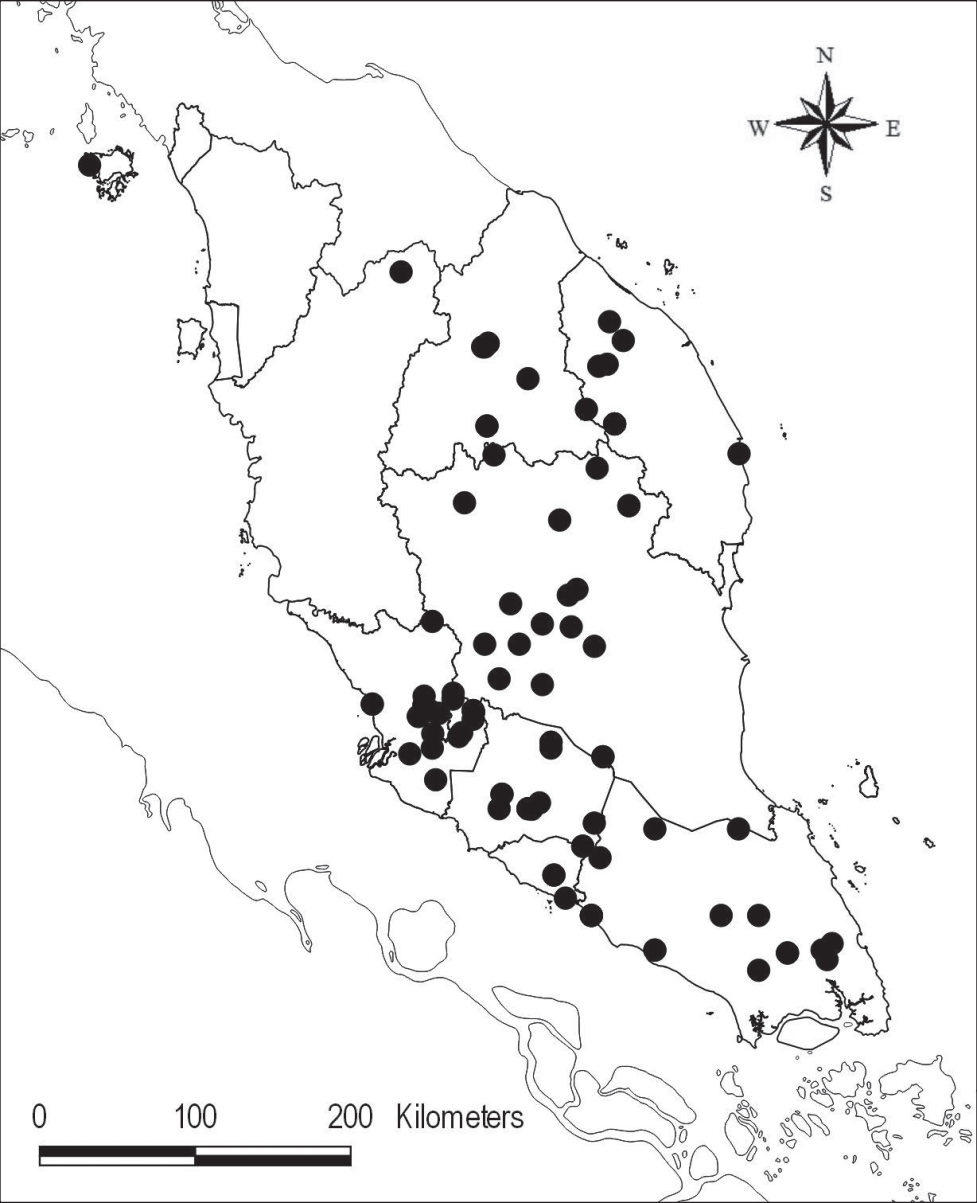
Distribution of *Chroesthes longifolia* (●) in Peninsular Malaysia.

Malaysia. Endemic to Peninsular Malaysia: Kedah, Perak, Kelantan, Terengganu, Pahang, Selangor, Negeri Sembilan, Melaka and Johor (Map 2).

#### Ecology.

In primary lowland forest up to upper hill dipterocarp forest at 60 to 700 m a.s.l. Sometimes also found in logged and disturbed forests.

#### Conservation status.

Least concern (LC). This is a widespread species throughout Peninsular Malaysia. However, many old collections are from places that have already changed its habitat. Development of these areas has led to population declines. However, its occurrence still has a wide range of distribution and many are still in totally protected areas.

#### Specimens examined.

**Peninsular Malaysia. Johor**: Batu Pahat: 6 November 1892, *Lake HW* s.n. (SING); *Ibid.*, November 1900, *Ridley HN* 11127 (SING); Johor Bahru: Sedenak, August 1908, *Ridley HN* 13496 (SING); Kluang: 16 November 1922, *Holttum RE* SFN9268 (SING); Kluang FR, 28 August 1950, *Sinclair J* SFN38950 (SING); Kota Tinggi: Gn. Panti, 23 June 1963, *Burkill HM* HMB 3178 (SING); Mawai, 9 September 1934, *Corner EJH* s.n. (SING); Mawai Rd., 24 January 1961, *Sinclair J* 10563 (SING); Sg. Linggiu, 26 July 1991, *Tay EP* 91-0052 (SING); Sg. Linggiu, 26 July 1991, *Tay EP* 91-52 (KEP); Muar: Bkt. Keyara, 1902, *Fox W* 11283 (SING); Gn. Ledang, June 1892, *Ridley HN* s.n. (SING); *Ibid*., Muar, April 1901, *Curtis C* s.n. (SING); Segamat: Sg. Juasseh, 28 June 1970, *Samsuri A* SA304 (SING); **Kedah**: Langkawi, Gn. Machinchang FR, 17 March 1969, *Chan YC* FRI11209 (SING); **Kelantan**: Gua Musang, 14 July 1935, *Henderson MR* SFN29657 (KEP,SING); Gua Musang: Relai FR, 1 September 1992, *Hamid* H2 (KEP); Kuala Krai: Gn. Stong, 5 March 1924, *Mhd Nur* SFN12180 (SING); Stong FR, 31 March 2009, *Rosdi M* FRI66253 (KEPSING); Stong Tengah FR, 6 February 2007, *Chew MY* FRI53481 (KEP); Kuala Lumpur, February 1890, *Curtis C* 2362 (SING); **Melaka**: Jasin: Air Panas, 5 September 1886, *Watchman under Derry* 667 (SING); Air Panas, November 1893, *Goodenough JS* 1690 (SING); Chabau, 22 September 1885, *Alvins MV* 2256 (SING); Merlimau, June 1889, *Derry R* 221 (SING); **Negeri Sembilan**: Jelebu: Pasoh FR, 3 June 1987, *LaFrankie JV* LJV2276 (KEP); *Ibid*., 4 July 1982 *Kiew R* RK1186 (KEP); *Ibid*., 30 August 2010, *Yao TL* FRI65469 (KEP); Kuala Pilah: Angsi FR, Gn. Angsi, 10 December 1930, *Syed A* FMS23764 (KEP, SING); Senaling, 24 June 1930, *Corner EJH* s.n. (SING); Senaling Inas,18 November 1929, *Symington CF* FMS 21356 (KEP, SING); Senaling Inas FR, 28 November 1922, *Holttum RE* 9788 (SING); Rembau: Perhentian Tinggi, December 1898, *Ridley HN* s.n. (SING); **Pahang**: Bentong: Clough FR, Cpt. 37, 16 July 1958, *Kochummen KM* KEP78709 (KEP); Bentung, Karak FR, 14 July 1924, Best, GA, SFN13899 (SING); Jerantut: Gn. Aais FR, 5 July 2004, *Sam YY* FRI49062 (KEP); *Ibid*., 6 July 2004, *Chua LSL* FRI46691 (KEP); Kota Gelanggi, August 1891, *Ridley HN* 2174 (SING); P. Tawar, July 1891, *Ridley HN* s.n. (SING); Taman Negara Gn. Tahan, 31 July 1996, *Kiew R* RK3978 (KEP); Taman Negara, Sg. Riul, 12 July 1970, *Everett B* FRI14436 (KEP); Taman Negara Ulu Sat Tg. Petir, 12 July 1970, *Whitmore TC* FRI15267 (KEP); Taman Negara Kuala Keniam, 7 September 2020, *Siti Munirah MY* FRI94881 (KEP); Lipis: Chegar Perah,14 October 1927, *Henderson MR* SFN19364 (SING); Maran: Jengka FR, 10 April 2001, *Sam YY* FRI 46518 (KEP); Temerloh: Gn. Benom, 14 June 1968, *Teo LE* 2714 (SING); Temerluh: Fort Iskandar, 2 March 1950, *Woods MC* KL1720 (SING); *Ibid*., 2 March 1959, *Woods MC* KL1720 (KEP); Gn. Senyum, 30 July 1928, *Henderson MR* s.n. (SING); Kemasul FR, 16 September 2006, *Hamid* FMS10598 (KEP); Krau WR, 10 November 1999, *Damahuri S* FRI45314 (KEP); Krau WR 12 July 2007, *Mohd Hairul MA* FRI58921 (KEP); **Perak**: Hulu Perak: Belum FR, 30 December 1993, *Davison GWH* UPM 6077 (KEP); **Selangor**: Gombak: Bkt. Lagong FR, 5 May 1976, *Chan YC* FRI23956 (KEP, SING); *Ibid*., 11 November 1959, *Kochummen KM* KEP94047 (KEP, SING); Kanching FR, 17 September 1925, *Strugnell EJ* FMS10508 (KEP); Kepong, 27 May 1970, *Teo LE* 2862 (SING); Ulu Gombak, 4 July 1918, *Sanger-Davies AE* FMS2376 (KEP, SING); Hulu Langat: Bkt. Batu Balai, 1 August 1959, *Gadoh U* KL1630 (KEP); Bkt. Enggang, 3 April 1930, *Symington CF* FMS 24134 (SING); Bkt. Tangkol, 1 October 1959, *Gadoh U* KL1810 (KEP); Bkt. Tangkol, 25 July 1959 *Gadoh U* KL1621 (KEP); Ulu Langat, 7 June 1979, *Rajoo* SA237 (KEP); Hulu Selangor: Gading Forest Reserve, 19 July 1969, *Chan YC* FRI11235 (KEP); Klang, Klang Water Catchment Forest, 12 March 1922, *Burkill IH* SFN6849 (SING); Petaling: Air Hitam Forest Reserve, 12 September 1985, *Kiew R* RK2065 (KEP); Petaling Rd., 21 June 1889, *Ridley HN* s.n. (SING); Sungai Buloh Forest Reserve, 7 October 1926, *Strugnell EJ* 12495 (KEP); Sungai Buloh Forest Reserve, 13 August 1923, *Hamid* FMS 8888 (KEP); Labu River, *Ridley HN* s.n. (SING); Sungai Buloh, 12 December 1923, *Mhd Nur* SFN11886 (SING); Sungai Buloh, 23 November 1956, *Burkill HM* HMB (SING); **Terengganu**: Dungun: Bukit Bauk, 25 January 1994, *Kiew R* RK3793 (KEP); Hulu Terengganu: Sg. Petuang, 26 March 1974, *Ng FSP* FRI22026 (KEP); Tasik Kenyir, 3 August 2007, *Kamarul Hisham M* FRI52138 (KEP, SING); Tasik Kenyir, 3 August 2007 *Julius A* FRI56144 (KEP); Tembat Forest Rerserve, 6 April 2009, *Rosdi M* FRI66281 (KEP); Tembat Forest Reserve, 3 April 2009, *Siti Munirah MY* FRI67889 (KEP); Setiu: Ulu Setiu Forest Reserve, 7 March 2002, *Saw LG* FRI44390 (KEP); Nerus Forest Reserve, Gunung Sarut, 24 June 2019, *Imin K* FRI 94046 (KEP).

#### Notes.

Ridley (1923) mentioned *Chroesthes macrantha* as present in Peninsular Malaysia based on Wray 3385 from Perak and that it is similar to *C. longifolia*. Hansen (1983) suggested that *C. macrantha* was probably a form of *C. longifolia*, but he had not observed Wray 3385, so was not able to clarify its status. Efforts to search for this specimen have also been unsuccessful. To date, the status of this taxon is uncertain. Besides, this name also appears not to have been validly published.

**Figure 8.**
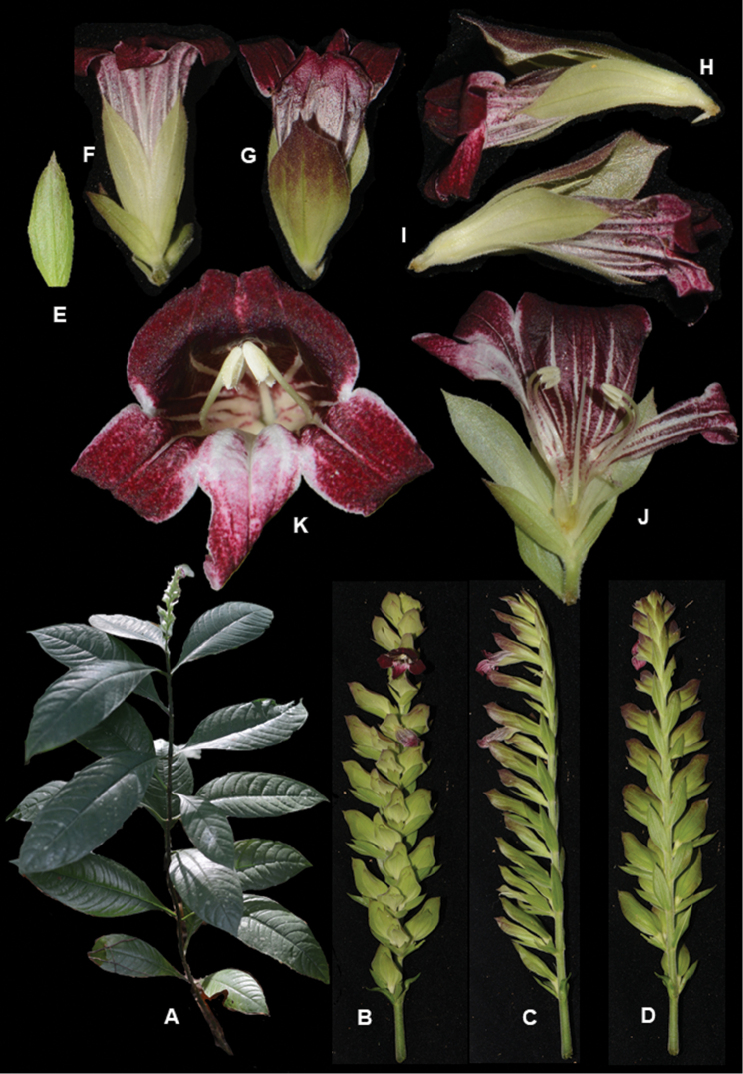
*Chroesthes longifolia* (Wight) B. Hansen, collected from Taman Negara, Pahang (Kuala Keniam) **A** habit **B–D** inflorescence **B** front view **C** side view **D** back view **E** bract **F–I** flower with bracteoles and calyx **F** from the top view **G** lower view **H–I** side view **J** corolla (inner view) showing the stamens and pistil **K** corolla lips (flower from front view) showing anthers (All photos by Siti-Munirah MY).

## Supplementary Material

XML Treatment for Chroesthes

XML Treatment for Chroesthes faizaltahiriana

XML Treatment for Chroesthes lanceolata

XML Treatment for Chroesthes longifolia
